# Patterned injuries from screwdrivers

**DOI:** 10.1007/s12024-022-00489-y

**Published:** 2022-06-15

**Authors:** Roger W Byard

**Affiliations:** 1 Forensic Science SA, Adelaide, Australia; 2 School of Biomedicine, The University of Adelaide, Level 2, Room N237, Helen Mayo North, Frome Road, Adelaide, 5005 SA Australia

**Keywords:** Screwdriver, Phillips head, Slotted, Death, Stabbing, Cruciate pattern

## Abstract

A 40-year-old man was stabbed multiple times with a cross-tipped, Phillips head screwdriver with many of the puncture wounds characterized by a cruciate pattern consistently measuring approximately 5 × 5 mm corresponding to the shape of the weapon. Death was due to a single penetrating wound to the chest that had transfixed the aorta. This report characterizes the features of injuries that may be inflicted by Phillips head screwdrivers, contrasting this with injuries that may occur if the weapon is a flat or slotted head screwdriver. Given their ready availability, ease of handling, and sharpness, screwdrivers are surprisingly rarely used in fatal assaults.

## Case report

A 40-year-old man had been stabbed multiple times by an assailant at his front door. He had collapsed inside the door and was unable to be resuscitated. A Phillips head screwdriver was subsequently found underneath the body. At autopsy 39 groups of injuries were identified with the minimal number of separate puncture wounds which would have been inflicted by separate blows being approximately 54. The most significant injury was a penetrating wound of the mid-anterior chest located in the midline approximately 85 mm below the jugular notch which was associated with a fracture of the sternum, a through-and-through 10-mm slit-like penetrating wound of the aorta with surrounding interstitial hemorrhage, a 150 ml hemopericardium, and bilateral 100 ml hemothoraces. It measured approximately 50 mm in length. This was the lethal injury which also involved mediastinal soft tissue trauma and a penetrating wound of the pericardial sac with a non-penetrating wound of the right ventricle.

**Fig. 1:**
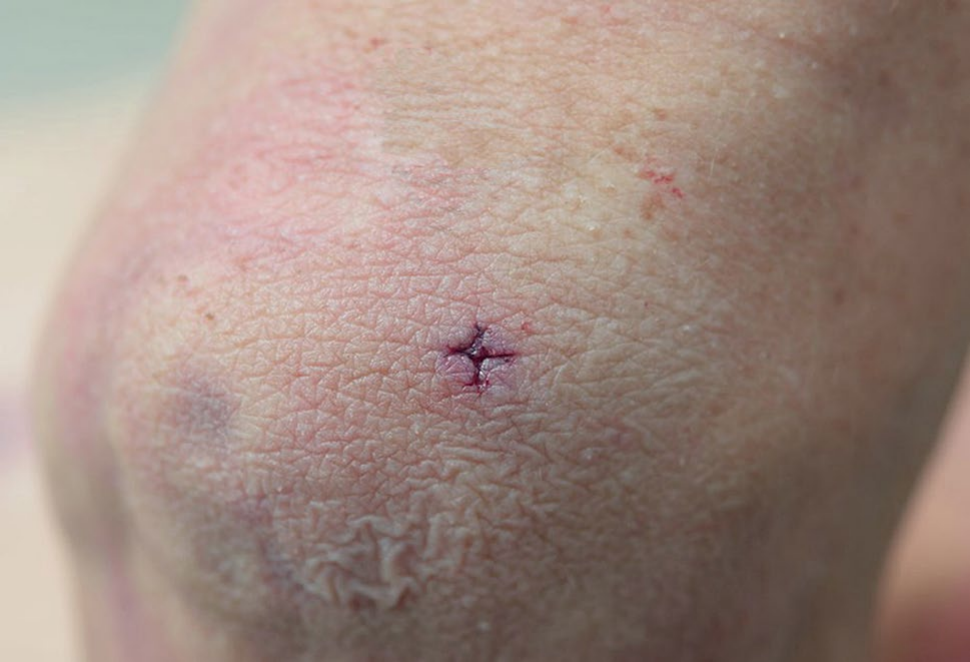
The left elbow of a 40-year-old man who had been stabbed multiple times with a cross-tipped, Phillips head screwdriver showing a characteristic cruciate cross-section measuring approximately 5 × 5 mm

**Fig. 2:**
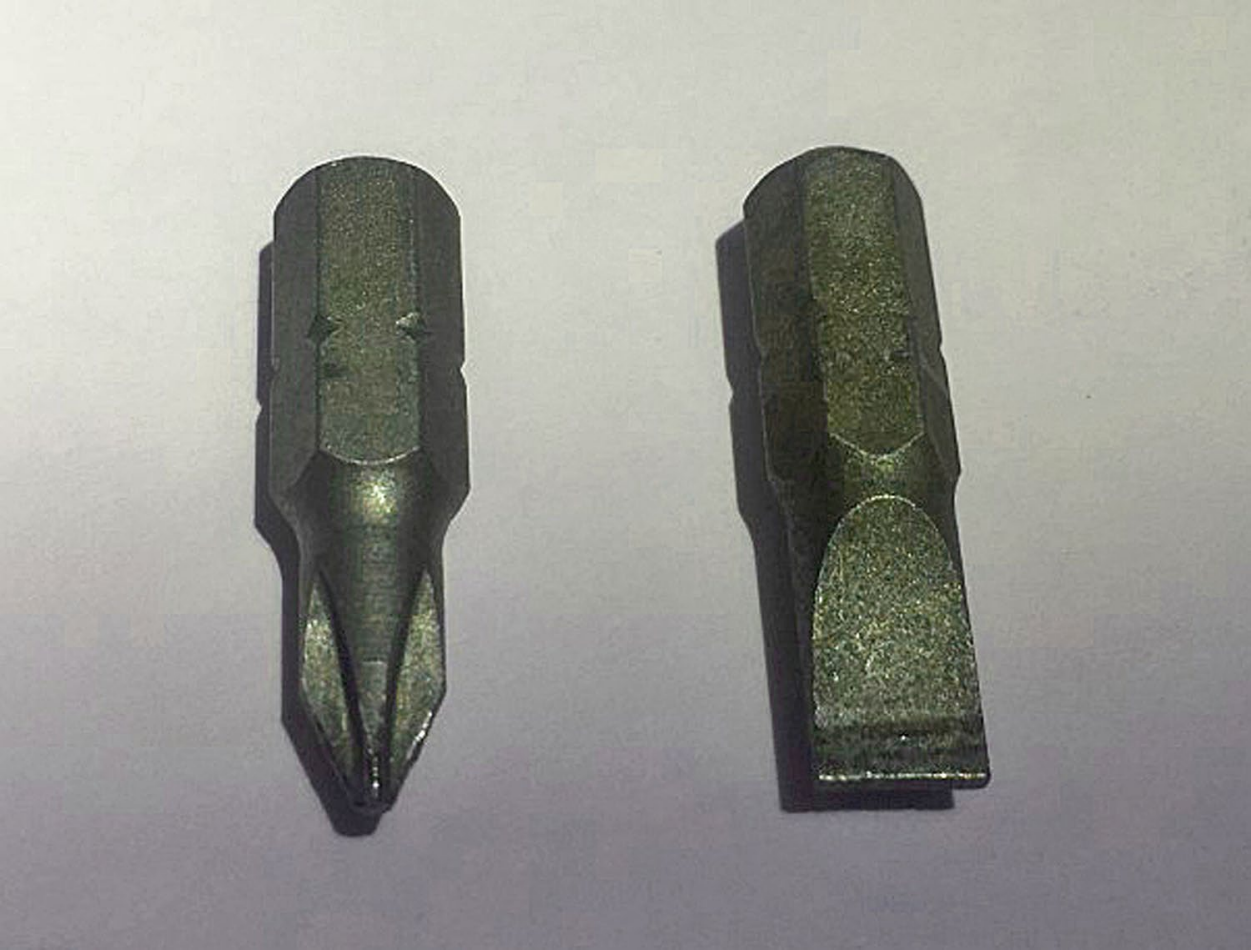
A typical Phillips head screwdriver with a cruciate cross-section contrasting with the rectangular profile of a flat or slotted head screwdriver

**Fig. 3:**
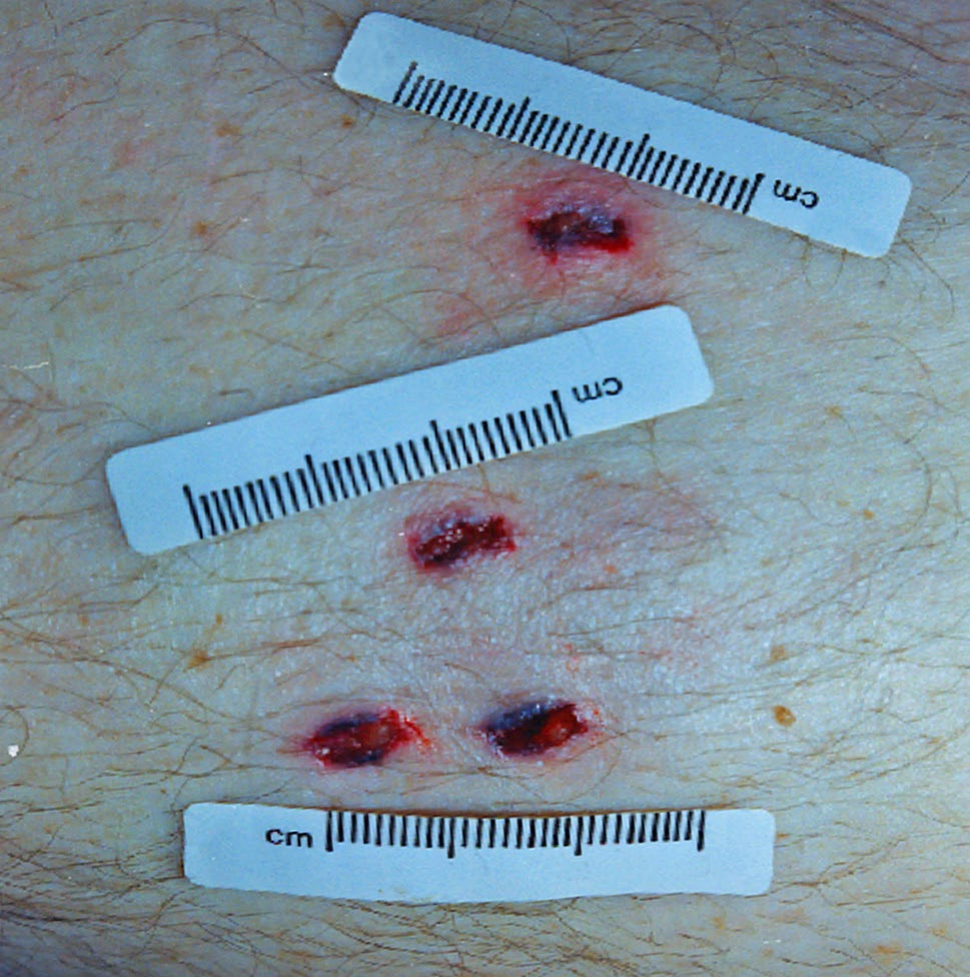
A cluster of four puncture wounds inflicted by a flat or slotted head screwdriver showing slightly irregular rectangular wounds with no tissue bridging but with subtly split edges and marginal abrasions/bruises

Other significant injuries included two puncture wounds of the right side of the mid back with penetration of the right lower lobe of the lung and a penetrating wound of the right eye with periorbital hemorrhage (“black eye”), a fracture of the right orbital plate and penetrating injuries of the right inferior gyrus rectus, right inferior genu corpus callosum, left inferior gyrus rectus, and left superior corpus striatum with extension into the left frontal horn. These were not associated with significant intracranial hemorrhage. Penetrating wounds were also present on the left side of the head with a small depressed fracture of left parietal bone and multiple superficial and penetrating wounds of the arms, anterior and posterior chest, neck, head, and face. The most striking feature of these injuries was that 34 of the puncture wounds demonstrated a cruciate pattern consistently measuring approximately 5 × 5 mm (Fig. 1) corresponding to the shape of a Phillips head screwdriver, contrasting to a flat or slotted head screwdriver (Figs. 2 and 3). The latter image (Fig. 3) was used for comparison purposes from a deidentified case taken from the Forensic Science SA archives.

**Fig. 4:**
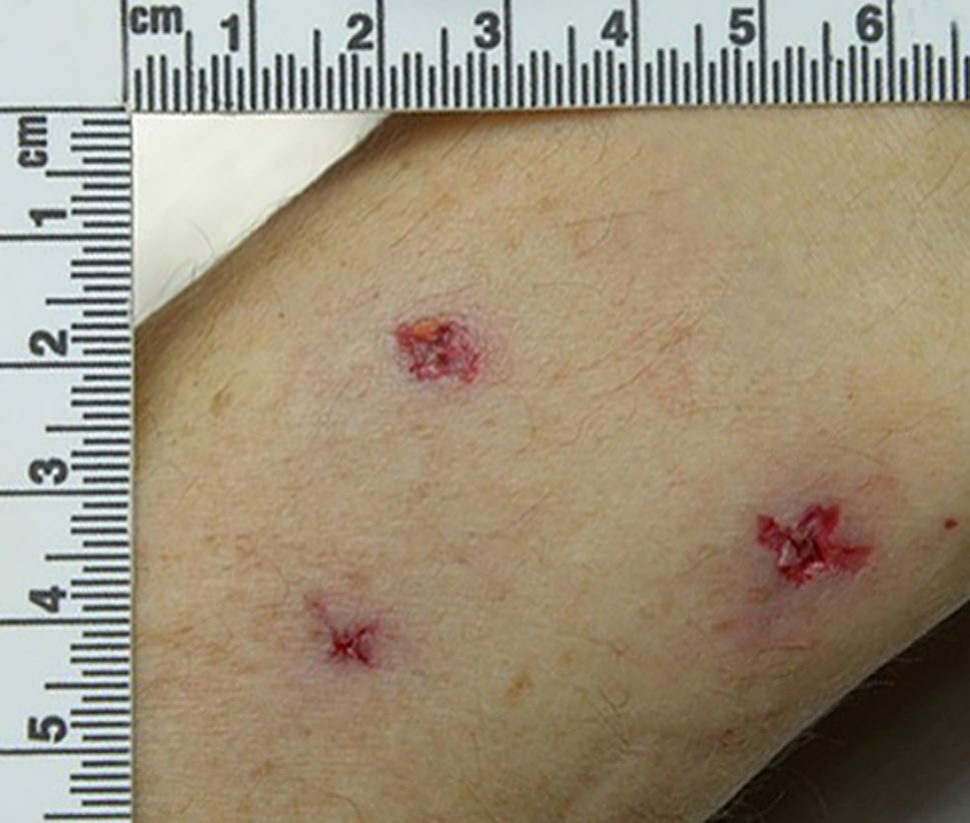
A grouping of three cruciate puncture wounds on the posterior aspect of the right wrist from the Phillips head screwdriver used in the reported case

**Fig. 5:**
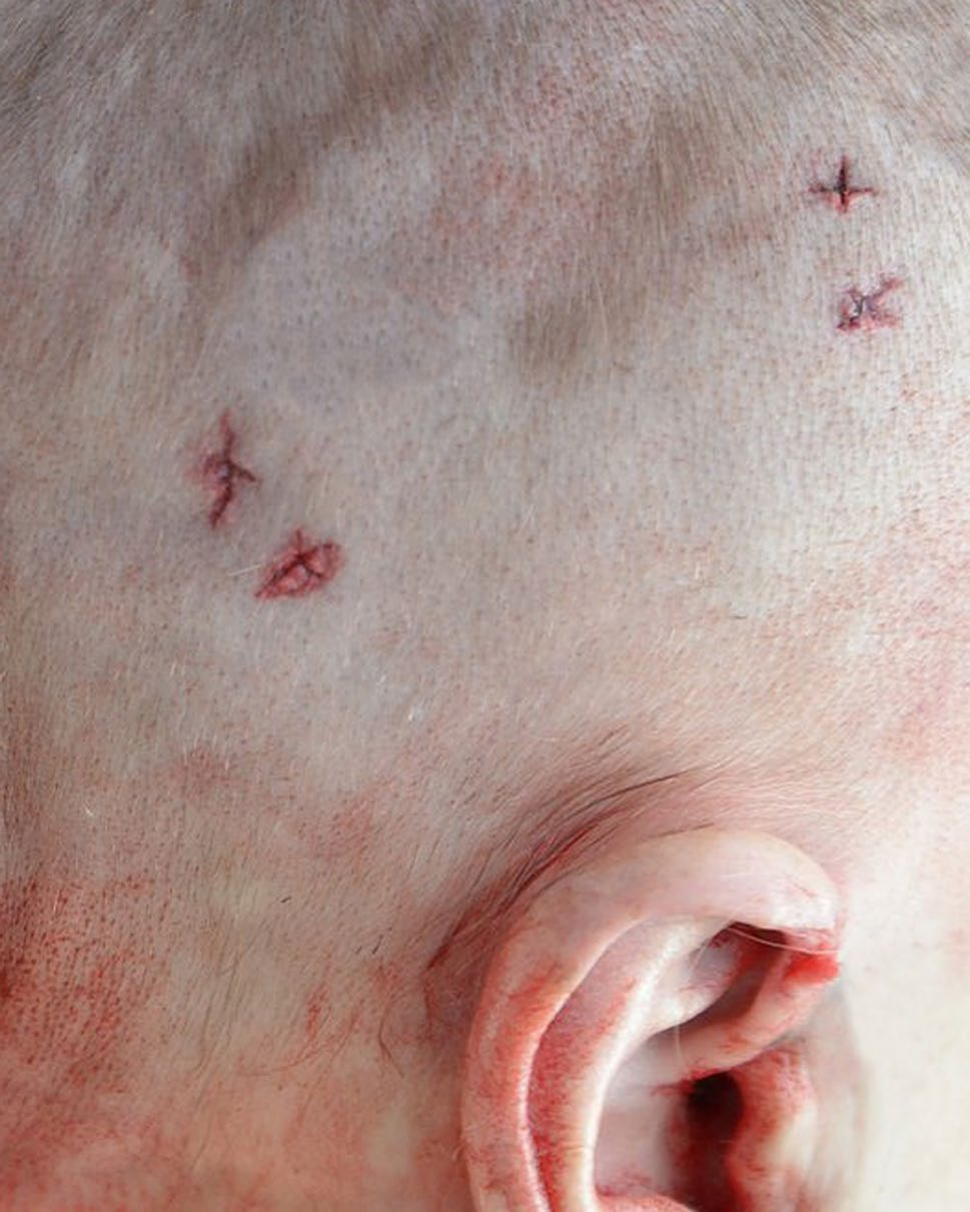
Two pairs of cruciate puncture wounds above the right ear

**Fig. 6:**
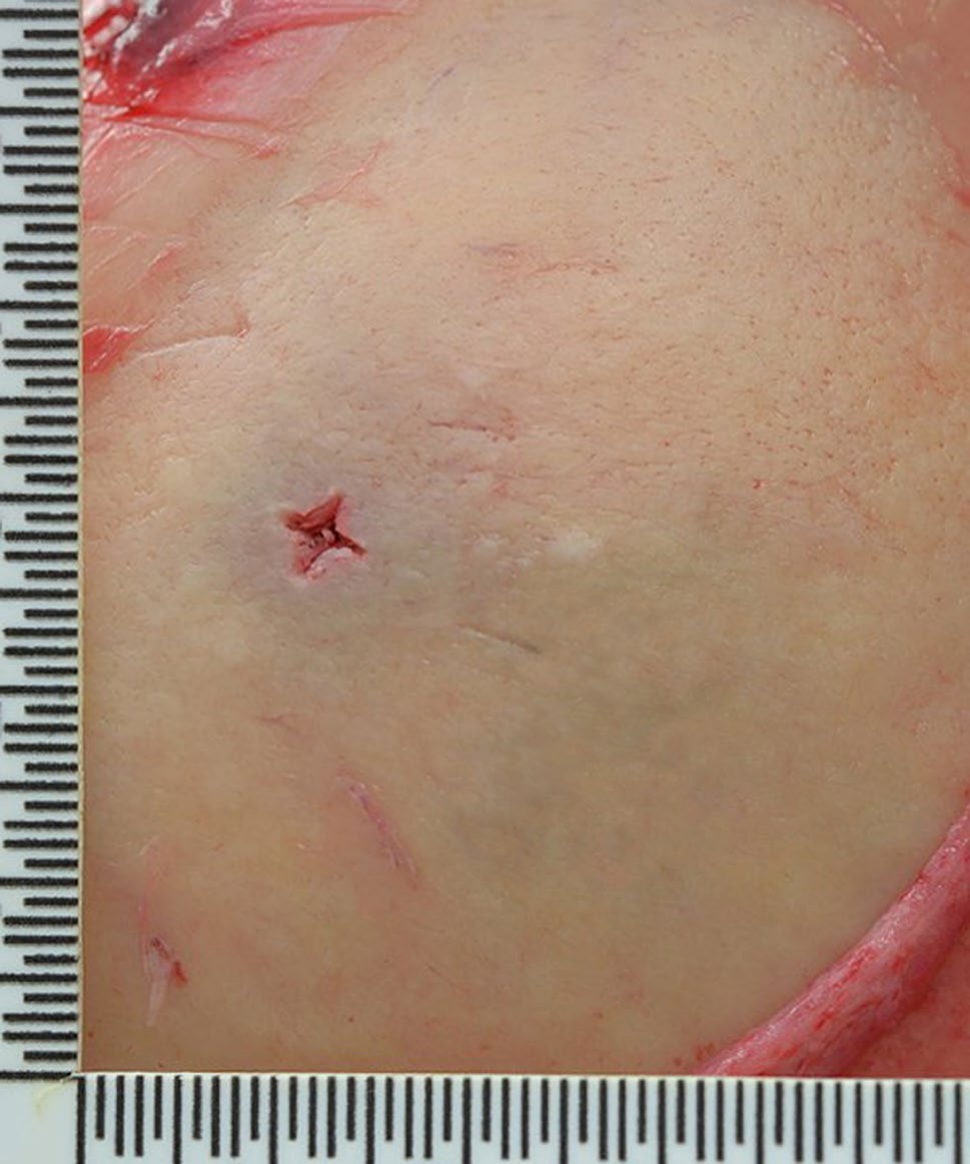
A cruciate wound in the left posterior parietal bone measuring 5 × 5 mm associated with an underlying 10-mm circular depressed fracture

Further examples of characteristic patterned injuries from the Phillips head screwdriver include a grouping of three cruciate puncture wounds on the posterior aspect of the right wrist (Fig. 4), two pairs of cruciate puncture wounds above the right ear (Fig. 5), and a cruciate wound in the left posterior parietal bone (Fig. 6). The absence of hilt marks may have been due to the weapon not being inserted to its full length or to the interposition of clothing and hair.

## Discussion

Screwdrivers can be used to inflict deep puncture wounds that may penetrate bone, including the skull, and cause lethal organ injuries. Although it has been suggested that screwdrivers cause typical incised wounds [1], this depends greatly on the sharpness of their tips. If, for example, the head of a screwdrivers is blunt, it may cause hybrid sharp-blunt force trauma wounds, as can be seen in Fig. 3 where the four closely placed wounds from a flat head screwdriver show slightly irregular central stab wounds with no tissue bridging but with subtly split edges and marginal abrasions/bruises. The Phillips head screwdriver in the reported case was sharper and so caused more typical sharp force injuries.

Screwdrivers have been rarely reported in the literature in cases of fatal assaults that have most often involved penetrating trauma to the torso or head [2, 3]. A screwdriver may be favored more in countries such as Greece where the possession of a concealed knife is illegal, but not a screwdriver [4]. Such non-projectile penetrating cerebral trauma is rare accounting for only about 0.4% of all head injuries [5, 6] and has involved a variety of objects including crochet hooks, a toilet brush handle, an antler, scissors, knitting needles, crossbow bolts, glass, car antennas, crowbars, pitchforks, a chair leg, and umbrella ribs [4, 7–10]. In attacks with screw drivers, the skull is usually penetrated in areas such as the orbital plates (as in the reported case) and the nasal or squamous temporal bones where the bone is thin [4, 6].

This case demonstrates a very graphic recording on the victim’s body of the exact nature of the weapon that was used in the assault. In cases where a weapon has been removed from the scene, this may be an extremely useful finding in focusing investigations on looking for a screwdriver rather than another type of sharp object.
